# Designing a 3D Printing Based Auxetic Cardiac Patch with hiPSC-CMs for Heart Repair

**DOI:** 10.3390/jcdd8120172

**Published:** 2021-12-03

**Authors:** Olga Brazhkina, Jeong Hun Park, Hyun-Ji Park, Sruti Bheri, Joshua T. Maxwell, Scott J. Hollister, Michael E. Davis

**Affiliations:** 1 Wallace H. Coulter Department of Biomedical Engineering, Georgia Institute of Technology and Emory University, Atlanta, GA 30332, USA; olga.brazhkina@emory.edu (O.B.); hyunji.park@emory.edu (H.-J.P.); srutibheri@gatech.edu (S.B.); 2 Center for 3D Medical Fabrication, Wallace H. Coulter Department of Biomedical Engineering, Georgia Institute of Technology and Emory University, Atlanta, GA 30332, USA; jeonghun.park@bme.gatech.edu; 3 Children’s Heart Research & Outcomes (HeRO) Center, Children’s Healthcare of Atlanta & Emory University, Atlanta, GA 30332, USA; joshua.t.maxwell@emory.edu; 4 Division of Pediatric Cardiology, Department of Pediatrics, Emory University School of Medicine, Atlanta, GA 30332, USA

**Keywords:** cardiac patch, auxetics, 3D printing, cardiomyocytes, myocardial infarction

## Abstract

Myocardial infarction is one of the largest contributors to cardiovascular disease and reduces the ability of the heart to pump blood. One promising therapeutic approach to address the diminished function is the use of cardiac patches composed of biomaterial substrates and cardiac cells. These patches can be enhanced with the application of an auxetic design, which has a negative Poisson’s ratio and can be modified to suit the mechanics of the infarct and surrounding cardiac tissue. Here, we examined multiple auxetic models (orthogonal missing rib and re-entrant honeycomb in two orientations) with tunable mechanical properties as a cardiac patch substrate. Further, we demonstrated that 3D printing based auxetic cardiac patches of varying thicknesses (0.2, 0.4, and 0.6 mm) composed of polycaprolactone and gelatin methacrylate can support induced pluripotent stem cell-derived cardiomyocyte function for 14-day culture. Taken together, this work shows the potential of cellularized auxetic cardiac patches as a suitable tissue engineering approach to treating cardiovascular disease.

## 1. Introduction

Myocardial infarction (MI) is a leading contributor to worldwide morbidity and mortality, which is associated with weakening of the left ventricle, leading to heart failure [1]. MI is caused by occlusion of the coronary arteries, resulting in disruption of nutrient supply and death of the contractile myocardium [1]. Following MI, the ventricular walls undergo intensive remodeling of the infarcted area, resulting in thinning, fibrosis, and reduced cardiac output [2,3,4]. This process alters the heart microenvironment, which cannot be reversed by generation of new contractile tissue [2,3,4,5]. These changes elicit major consequences on the geometry and function of the heart, often causing permanent adverse remodeling. Treatments for MI have been widely investigated, including the application of cardiac cell therapies and cardiac patches [4,6,7,8,9]. However, progression to the clinic has been limited by poor engraftment associated with cell therapies, as well as inappropriate biological cues [6,7,8].

Biomaterials have been used as patch substrates due to tunable degradation rates, while also allowing tailored mechanical properties to potentially match the ventricle mechanical properties during the extensive remodeling process [9,10,11]. Cardiac patches have been made from a variety of combinations of cell sources, biological molecules, and both natural and synthetic biomaterials, with designs aimed at regulating cellular responses in the infarct and mimicking the physiology and mechanics of the heart [4,9]. With the focus on global mechanics of the left ventricle, cardiac patches are typically matched to the heart’s tangent Young’s modulus, specifically one that is physiologically healthy [10,12]. Infarcted tissue usually becomes stiffer, and the tissue stiffness evolves through the duration of the remodeling to improve pumping ability after MI [2,3,4]. Additionally, the healthy heart nonlinear elastic mechanical properties are anisotropic, and are therefore dependent on orientation on the ventricle. These complex nonlinear mechanical properties depend significantly on patient factors, including age and overall cardiac health [10,11,12,13,14]. As cardiac patch materials are selected to provide mechanical unloading on the infarct and alter extracellular matrix (ECM) deposition and metabolism during remodeling, focusing on matching only one infarcted parameter or the global ventricular mechanics does not accurately integrate the complexity of infarct region into the design. A cardiac patch designed to possess tunable properties based on local mechanics of the infarcted region could provide enhanced mechanical support of the ventricle for the duration of remodeling.

One of the main design criteria for cardiac patch research has been mimicking cardiac mechanical properties, either though bulk material characteristics, orientation, or fiber alignment [4,10,12,15]. An underexplored aspect to address these parameters is applying auxetic designs, as compared to conventional materials in patterned and unpatterned approaches. A previous study examining a conductive auxetic patch demonstrated a greater confirmation to local mechanics over non-auxetic patches [16]. Auxetic materials describe a class of materials that possess a negative Poisson’s ratio, as they are able to expand in multiple directions when stretched longitudinally [17,18,19,20]. Auxetic patterns have high shock and energy absorption and are resistant to fracture under repeated loading conditions, making them desirable for cardiac applications [19,20]. Incorporation of auxetic designs can improve a material’s shear resistance, indentation resistance, and synclastic curvature behavior [16,19]. In addition to tunable strength, these auxetic design qualities are favorable for cardiac patches to address the elevated degree of complexity associated with infarct remodeling. A recent study demonstrated that an auxetic patch design provides greater conformity to local mechanics of native rat left ventricular tissue over a non-auxetic patch, while both groups demonstrated global mechanical support [16]. The study highlights how accounting for the mechanics of the infarcted area, not just the surrounding healthy tissue, is crucial for providing mechanical support for the ventricle wall.

This work seeks to develop a 3D printing based auxetic cardiac patch that could be therapeutically used to improve cardiac function post MI. The cardiac patch substrate was designed from auxetic units and further optimized to potentially overcome limitations of previous cardiac patches with tunable stiffness and isotropic behavior to simulate rat infarct mechanics. We further demonstrated that a 3D printing based auxetic cardiac patch composed of auxetic polycaprolactone (PCL) substrate and gelatin methacrylate (GelMA) is capable of supporting human induced pluripotent stem cell-derived cardiomyocytes (iPSC-CMs) as a potential therapy. A closer investigation into regeneration or repair of infarcted tissue using auxetic cardiac patches with adjustable mechanical properties could lead to the development of new tissue engineering approaches to treating MI and other cardiac diseases.

## 2. Materials and Methods

### 2.1. Design and Finite Element Analysis of Auxetic Patch Substrates

Finite element analysis (FEA) was performed using FEBio Studio (version 1.5, Febio.org, University of Utah, Salt Lake, UT, USA) to compare the effective mechanical properties and behavior of two auxetic patches under uniaxial tension [21,22]. The base PCL of auxetic patches was modelled as a linear isotropic elastic material with a Young’s modulus of 0.192 GPa and Poisson’s ratio of 0.3. The Young’s modulus of PCL was obtained from the tensile tests using ASTM D638 Dumbbell shape specimens on a universal mechanical testing machine (3340 Series Single Column Systems; Instron, Norwood, MA, USA) equipped with a 2 kN load cell.

### 2.2. 3D Printing of Auxetic Substrate Framework

A customized extrusion-based 3D printing system (developed in collaboration with T&R Biofab Co., Ltd., Si-heung, Korea), was used for printing of the missing rib auxetic cardiac patches substrates. Poly-ε-caprolactone (PCL, CAPA 6501, M_w_ = 50,000; Polysciences Inc., Warrington, UK) was used for all printing of auxetic cardiac patch substrates by melting at 135 °C inside a stainless-steel syringe, and extruded through a 150 µm size nozzle. Auxetic cardiac patches were printed with different thicknesses according to a pre-defined printing pathway in G-code within a biosafety cabinet with 10 °C inner temperature.

### 2.3. Mechanical Testing

Printed orthogonal missing rib auxetic patches with and without GelMA were tested uniaxially in tension at 0.2 mm/min displacement rate. Load and displacement were monitored during the testing and tangent tensile moduli were calculated from the first linear region of the stress–strain curves (within 5% strain).

### 2.4. Human Induced Pluripotent Stem Cell Culture

For human induced pluripotent stem cell (hiPSCs; SCVI-113; Stanford, CA, USA) cultures, cells were maintained in 6-well tissue culture plates coated in hESC-qualified Matrigel Matrix (Corning, Corning, NY, USA) or LDEV-free reduced growth factor Geltrex Matrix (Thermo Fisher, Waltham, MA, USA) at 1:100 dilution with daily feeding with mTESR Plus (StemCell Technologies, Vancouver, BC, Canada) maintenance media. Upon reaching 70% confluency, cells were dissociated in PBS with 0.5 mM EDTA (Thermo Fisher) for 11 min at 37 °C and passaged at a 1:18 splitting ratio. First 24 h after replating included 10 µM of Y-27632 (StemCell Technologies, Vancouver, BC, Canada) in mTESR Plus media.

### 2.5. Human Induced Pluripotent Stem Cell-Derived Cardiomyocyte Differentiation

A differentiation protocol was adapted from previous work on modulation of Wnt signaling in iPSCs and subsequent glucose starvation [23,24,25,26]. Upon reaching 95–100% confluency, 3 mL/well of RPMI 1640 (Thermo Fisher) supplemented with B27 minus insulin (Thermo Fisher) with 5 µM CHIR 99,021 was added at day 0. On day 1, 2 mL/well RPMI 1640 with B27 minus insulin was added to existing media to create a concentration gradient between day 0–2. On day 2, media was replaced with RPMI 1640 with B27 minus insulin. On day 3, media was replaced to RPMI 1640 supplemented with B27 minus insulin with 10 µM IWR-1 (Sigma-Aldrich, St. Louis, MO, USA). On days 5–9, RPMI 1640 supplemented with B27 (Thermo Fisher) was used every other day. On day 10, media was changed to 3 mL/well RPMI 1640 minus glucose (Thermo Fisher) supplemented with B27 with 5 mM Sodium L-Lactate (Sigma-Aldrich) for glucose starvation to enhance cardiomyocyte purity and kept for 3 days. On day 13, cells were dissociated using STEMdiff Cardiomyocyte Dissociation Medium (StemCell Technologies) and replated at 2 million cells/well in 6-well tissue culture plates coated in hESC-qualified Matrigel Matrix (Corning) or LDEV-free reduced growth factor Geltrex Matrix (Thermo Fisher) at 1:60 dilution. For the first 24 h after replating, 2 mL/well of STEMdiff Cardiomyocyte Support Medium (StemCell Technologies) was added. On day 14, media was changed to 3 mL/well RPMI 1640 minus glucose supplemented with B27 with 5 mM Sodium L-Lactate for secondary glucose starvation. For hiPSC-CM culture, 2 mL/well of STEMdiff Cardiomyocyte Maintenance Kit (StemCell Technologies) was refreshed every other day.

### 2.6. Patch Fabrication and Cell Viability

Lyophilized GelMA (Cellink, Boston, MA, USA) was dissolved in PBS at 70 °C for 2 h with intermittent vortexing until fully dissolved. The crosslinking reagents included 0.01 mM Eosin Y (Santa Cruz Biotechnology, Dallas, TX, USA), 37.5 nM 1-vinyl-2-pyrrolidione (NVP; Sigma-Aldrich), and 0.1% *v*/*v* triethanolamine (TEOA; Sigma-Aldrich) and the solution was cooled to 37 °C [27,28,29]. On days 20–24 (passage 1) hiPSC-CMs at 100 million cells/mL cell seeding density were mixed in 100 µL of 10% *w*/*v* GelMA solution and used for immunofluorescent staining and intracellular Ca^2+^ transients. On day 15 (passage 0) hiPSC-CMs at 10 million cells/mL seeding density were examined for additional immunofluorescent staining, real-time qRT-PCR, and cell viability. The sterilized auxetic PCL substrate was placed in each well of a 6-well tissue coated plate, and the GelMA cell suspension was pipetted into the substrate. Next, the patches were crosslinked for 5 min under white light (Braintree Scientific, Braintree, MA, USA) to induce radical polymerization, washed 3 times with Dulbecco’s phosphate-buffered saline (dPBS, no calcium, no magnesium; Thermo Fisher), and cultured with STEMdiff Cardiomyocyte Maintenance Kit (StemCell Technologies), refreshed every other day. Bright-field images of all patches were performed at with an Olympus 1 × 71 Inverted Microscope (Olympus Corporation, Tokyo, Japan). Cytotoxicity was quantified using an LDH Cytotoxicity Assay Kit (Cayman Chemical Company, Ann Arbor, MI, USA) following manufacturer guidelines. Briefly, conditioned medium was collected from patches at day 1, 3, and 7 LDH reaction solution was applied in duplicate with additional medium-only and positive and negative cells only controls to a 96-well plate. Absorbance readings at 490 nm were recorded using a microplate reader (BioTek Synergy 2; Biotek, Winooski, VT, USA) and readings were normalized to the controls. Cell viability was quantified as percent cytotoxicity subtracted from 100%.

### 2.7. Immunofluorescence Staining

Human induced pluripotent stem cell-derived cardiomyocyte embedded patches were subjected to immunofluorescence staining at day 1, 7, and 14 after cell seeding. Briefly, fixed samples were permeabilized with 0.1% (*v*/*v*) Triton X-100 (Sigma-Aldrich) in PBS for 15 min, blocked with 4% normal goat serum (Abcam, Cambridge, UK) for 1 h, incubated with mouse anti-cardiac troponin T monoclonal antibody (1:300, Thermo Fisher Scientific) overnight at 4 °C and then incubated with Alexa Fluor 647 goat anti-mouse IgG antibody (1:200, Thermo Fisher Scientific) for visualization. The patches were also incubated with mouse anti-⍺-actinin antibody (1:100, Millipore-Sigma, Burlington, MA, USA) overnight at 4 °C and then incubated with Alexa Fluor 594 goat anti-mouse IgG antibody (1:200, Thermo Fisher Scientific) for visualization. Nuclei were counterstained with 4′,6-Diamidine-2′-phenylindole dihydrochloride (DAPI, Sigma-Aldrich) and examined with confocal microscopy (Fluoview 1000, Olympus Corporation, Tokyo, Japan).

### 2.8. Real-Time qRT-PCR

Cell-specific gene expressions were quantified by qRT-PCR analysis. Briefly, total RNA from hiPSC-CMs were isolated and collected using the RNeasy Mini kit (Qiagen GmBH, Hilden, Germany). The collected RNA samples were then reverse transcribed into cDNA with MJ Mini thermal cycler (Bio-Rad, Hercules, CA, USA) using PrimeScript 1^st^ strand cDNA synthesis kit (Takara Bio, Tokyo, Japan). To analyze cardiac-specific and pluripotent gene expressions in hiPSC-CMs, qRT-PCR was performed with a StepOnePlus RealTime PCR system (Applied Biosystems, Foster City, CA, USA) using TaqMan Fast Universal PCR Master Mix (Applied Biosystems). The target genes were assessed using commercially available primers (Cx43 (GJA1): Hs00748445_s1, TNNT: Hs00748445_s1, OCT4 (POU5F1): Hs00999632_g1, SOX2: Hs01053049_s1, NANOG: Hs02387400_g1; Applied Biosystems). The results were quantified by the comparative Ct method. Ct values for samples were normalized to the expression of the housekeeping gene, glyceraldehyde 3-phosphate dehydrogenase (GAPDH: Hs99999905_m1; Applied Biosystems).

### 2.9. Intracellular Ca^2+^ Measurements

Confocal microscopy (Fluoview 1000, Olympus Corporation, Tokyo, Japan) was used to image Ca^2+^ transients, with excitation at 488 nm and emission collected at >500 nm. Human induced pluripotent stem cell-derived cardiomyocytes embedded in patches were loaded with 10 µm fluo-4/AM for 20 min at room temperature, followed by a 20 min-wash in Ca^2+^-free Tyrode’s solution at room temperature. Calcium transient measurements were acquired from spontaneously beating iPSC-CMs perfused with Tyrode’s solution in line scan mode at 2 ms/line with a pixel size of 0.155 µm using the 40x objective. All fluorescent signals were background subtracted. Changes in [Ca^2+^]_i_ are expressed as ΔF/F0, where ΔF is the change in fluorescence (measured fluorescence [F]–F0) and F0 is resting baseline fluo-4 fluorescence. For all Ca^2+^ imaging experiments, gels were placed on glass coverslips. Experiments were conducted at room temperature. Transients were detected and analyzed using Clampfit 11.2 (Molecular Devices, San Jose, CA, USA).

### 2.10. Statistical Analysis

Statistical analysis was performed using GraphPad PRISM 8 software (GraphPad, San Diego, CA, USA) with additional details outlined in figure captions.

## 3. Results

### 3.1. Finite Element Anylysis of Auxetic Cardiac Patch Substrate Designs

**Figure 1:**
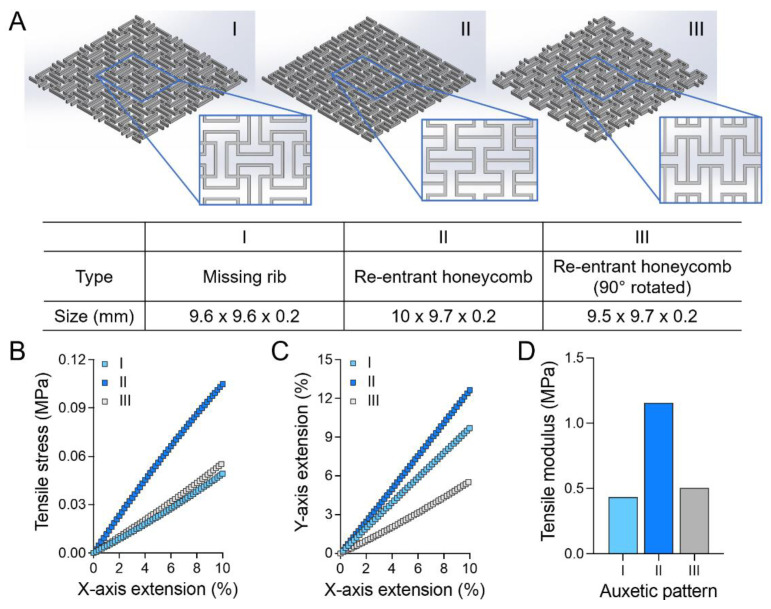
Finite element analysis of auxetic patches for the three different patterns. (**A**) Auxetic patch structures of orthogonal missing rib (**I**), 0° (**II**), and 90° (**III**) rotated re-entrant honeycomb; (**B**) Stress–strain and (**C**) X vs. Y extension curves of auxetic patches; (**D**) Tensile modulus of auxetic patches by patterns.

Two representative auxetic units, missing rib and re-entrant honeycomb, were used for cardiac patch substrate design (Figure 1A(I,II)). Both substrates have orthogonal patterns with approximately 10 × 10 mm^2^ size in area, with the same 100 µm line width, 300 µm line interval, and 200 µm thickness. In the missing rib design (I), symmetric square wave patterns along the *X*- and *Y*-axis were arranged and intersected. In the re-entrant honeycomb design (II), symmetric square wave patterns were also arranged along an axis and each pattern was connected through a parallel line. A third auxetic patch design was also explored (III), consisting of the re-entrant honeycomb rotated 90⁰ with the same parameters. In the simulation, orthogonal missing rib was extended only to the *X*-axis (group I, Figure 1A), as it is symmetric about the *X*- and *Y*-axis, while the orthogonal re-entrant patches were extended along the *X*- and *Y*-axis (group II and III, Figure 1A), respectively.

All auxetic substrates showed concurrent axial and transverse expansion under uniaxial tensile strain (Figure 1C). The re-entrant substrate showed variable perpendicular extension according to the tensile directions. The orthogonal missing rib patch (I) showed the smallest tensile modulus, comparable to the physiological range of human heart tissue tangent moduli (0.02–0.5 MPa), while the re-entrant patch (II) showed higher moduli than missing rib, which are different by tensile loading directions (Figure 1B–D) [10,11,12,13,14].

### 3.2. Mechanical Properties of Orthogonal Missing Rib Auxetic Patches

**Figure 2:**
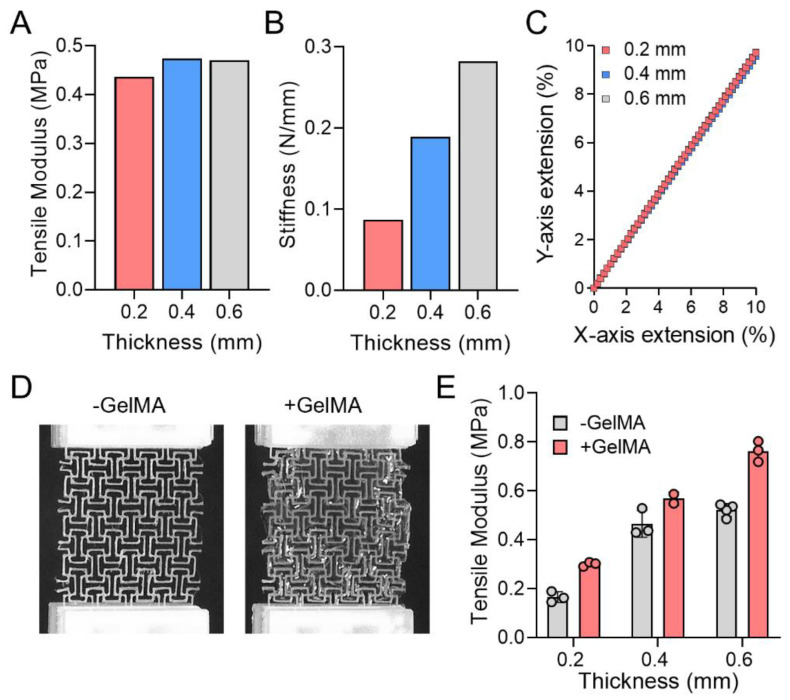
Mechanical properties analysis of orthogonal missing rib substrates according to the different thicknesses and GelMA application. (**A**) Tensile modulus of missing rib substrates by thickness; (**B**) Stiffness and (**C**) X vs. Y extension curves of missing rib substrates by thickness. (**D**) Tensile test and (**E**) modulus of missing rib substrates with and without GelMA application. Mean ± SD.

The orthogonal missing rib (group I) substrate had the smallest effective tensile modulus and was used for further FEA simulations to analyze the effect of substrate thickness on mechanical properties. Three thicknesses were chosen for the simulations: 0.2, 0.4, and 0.6 mm, with a fixed patch line width and interval. The simulations showed that all thicknesses have similar tensile moduli, while geometric stiffness increased with increasing thickness. The perpendicular extension to the tensile direction was constant regardless of thickness (Figure 2A–C).

Orthogonal missing rib substrates were successfully created using extrusion-based 3D printing and used for tensile testing (Figure 2D). Gelatin methacrylate was added to the PCL substrates to examine effects on tensile modulus. Tensile tests demonstrated that the tensile modulus of 0.4 and 0.6 mm thickness samples were similar to FEA results, but the 0.2 mm thickness substrate showed lower tensile modulus than FEA results (Figure 2E). The application of GelMA increased the tensile modulus of the missing rib substrates, yet the modulus of 0.2 and 0.4 mm thickness remained within the tangent physiological range [10,11,12,13,14].

### 3.3. In Vitro Characterization of Induced Cardiomyocyte-Laden Auxetic Patches

**Figure 3:**
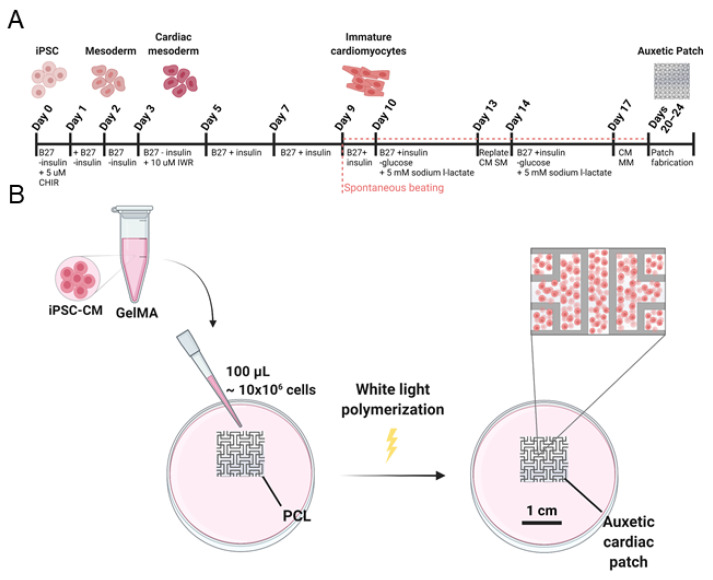
Cardiomyocyte incorporation into the auxetic patch. (**A**) Schematic timeline of Wnt-modulated cardiac differentiation and subsequent glucose starvation; (**B**) Fabrication process of auxetic cardiac patch composed of PCL and GelMA.

The auxetic substrates were evaluated for supporting cellular activity using induced pluripotent stem cell-derived cardiomyocytes (iPSC-CMs). First, we optimized established methods for iPSC differentiation into the cardiac lineage for our iPSC cell line (SCVI 113) and observed beating cardiomyocytes on day 9 (Figure 3A; Supplemental Video S1) [23,24,25,26]. Following glucose starvation in a lactate-supplemented media and replating in support medium (SM), the purified population of cardiomyocytes was supplemented with maintenance medium (MM) and used in downstream patch fabrication. Cardiomyocytes at a density of 100 million cells/mL were incorporated into GelMA, and then used to fill the 3D printed PCL substrate (Figure 3B). Eosin Y based white light crosslinking was used for radical polymerization to limit cell damage [27,28,29].

**Figure 4:**
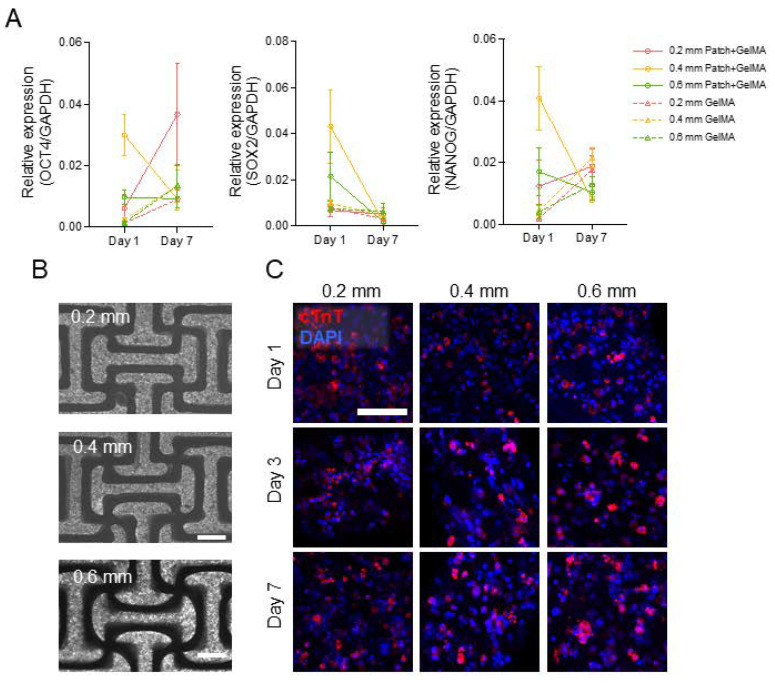
(**A**) Expression of pluripotent genes (Oct4, Nanog, and Sox2) from iPSC-CMs embedded in auxetic patches and GelMA alone patches, collected at day 1 and day 7. Mean ± SD. (**B**) Representative bright-field images of CM distribution in auxetic patches at day 7. Scale bar = 500 µm. (**C**) The auxetic patch embedded iPSC-CMs were stained against cardiac troponin T (red) and nucleus (blue) at day 1, 3, and 7. Scale bar = 100 µm.

After incorporating the cell suspension into the patches, we evaluated the gene expression of embedded induced cardiomyocytes against induced pluripotent genes (Oct4, Sox2, and Nanog), confirming low expression profiles for day 1 and day 7 cell patches relative to cell-only iPSCs (Figure 4A). These pluripotent genes expressions in embedded cardiomyocytes were less than 1/25 compared to iPSCs and further decreased in day 7. Next, we examined the effectiveness in creating homogenously distributed cardiomyocyte-laden patches of different thicknesses. Cells appeared evenly dispersed throughout the whole volume of all patch sizes and began beating after 24 h of culture (Figure 4B). Through the continued culturing of the patches, the induced cardiomyocytes migrated into clusters and showed minimal synchronous beating in their clusters (Supplemental Video S2). All cell cytotoxicity was examined LDH cytotoxicity assay (Supplemental Figure S1). The 0.6 mm patch thicknesses showed over 65% average viability after 7 days, with 0.4 mm patches showing over 75% average, and 0.2 mm thickness showing higher than 90% viability. To evaluate the cardiomyocyte organization within varied thicknesses of the patches, we performed immunofluorescence analysis for cardiac troponin T and *α*-actinin after 1, 3, and 7 days of culture for the three patch thicknesses (Figure 4C, Supplemental Figure S2). Staining showed presence of cardiac troponin T for 7-day culture conditions on all thicknesses. Minimal α-actinin is present on all thicknesses, which can be attributed to the immature phenotype of the induced cardiomyocytes. There was no observed functional spatial organization of cardiomyocytes by day 7, although minimal nuclei clustering is demonstrated.

**Figure 5:**
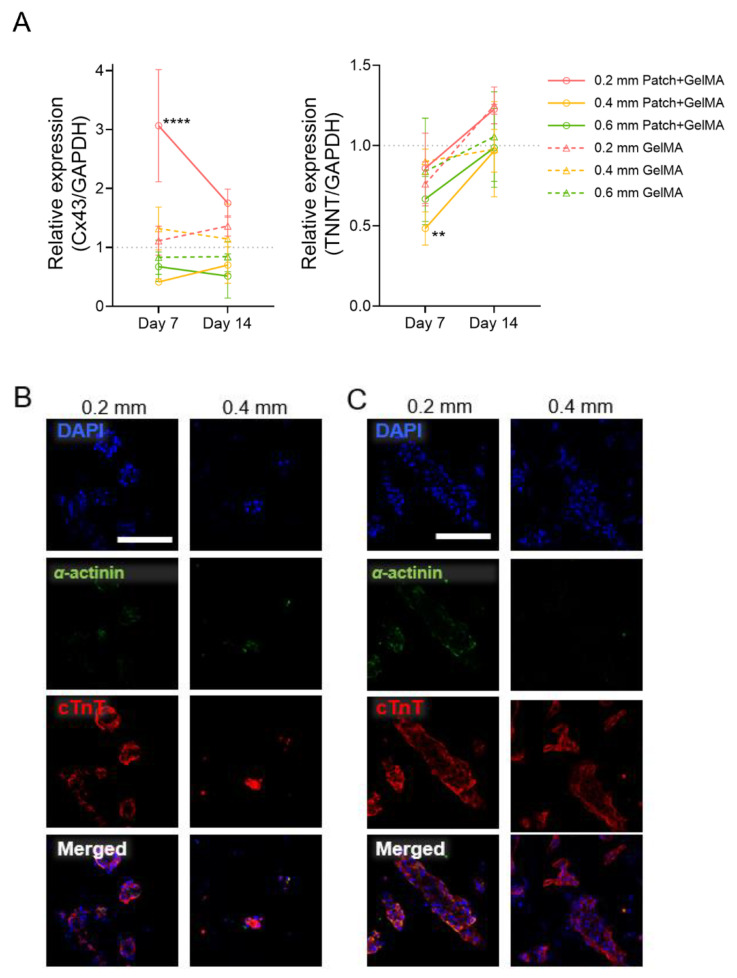
Examining cardiomyocyte morphology in patches at day 14. (**A**) Expression of cardiomyocyte-specific genes (Cx43 and TNNT) from cells embedded in auxetic patches and GelMA alone substrates, collected at day 7 and day 14. Mean ± SD. Significance was tested with two-way ANOVA with Dunnet multiple comparisons test, *n* = (3–4) for all samples at all time points. **** *p* < 0.0001 and ** *p* < 0.01 vs. iPSC-CMs at same day. (**B**) Low density and (**C**) high density iPSC-CMs in 0.2 and 0.4 mm patches were stained against cardiac troponin T (red), α-actinin (green), and nucleus (blue). Scale bar = 100 µm.

Gene expression for Cx43 (connexin 43) and TNNT (cardiac troponin T) was performed to examine the induced cardiomyocyte expression over the 14-day culture in the PCL and GelMA patches compared to 2D-cultured cardiomyocytes (Figure 5A). Both markers showed expression relative to the cell-only induced cardiomyocytes in both day 7 and day 14 after embedding. Especially, among six groups, cardiomyocytes embedded in 0.2 mm patch + GelMA showed higher Cx43 and TNNT expressions. Next, two induced cardiomyocyte seeding densities were investigated for spatial organization for a static 14-day culture. In addition to the higher density of 100 million cells/mL, induced cardiomyocytes at a lower density of 10 million/mL were incorporated into GelMA, and then used to fill 0.2 and 0.4 mm thicknesses of 3D printed PCL substrate, following previous methods. Immunofluorescent staining for cardiac troponin T and α-actinin displayed both groups of densities clustering of nuclei and spatial aggregation of the cells in the gel (Figure 5B,C).

**Figure 6:**
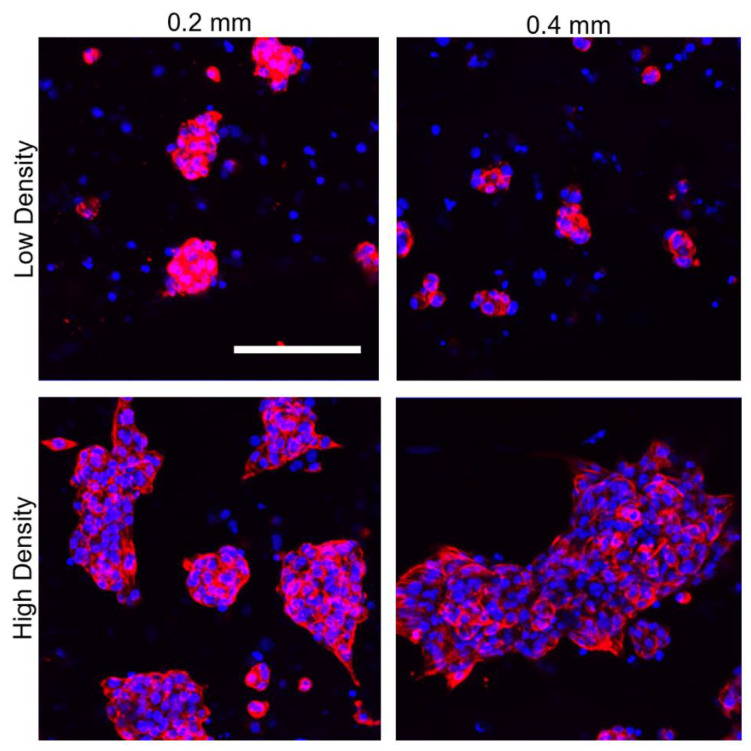
Maximum intensity projections from z-stacks of 0.2 and 0.4 mm high density and low density patches with iPSC-CMs stained against cardiac troponin T (red) and nucleus (blue) at day 14 of culture. Scale bar = 100 µm.

To further examine the cell clustering at day 14, z-stacks were acquired, and maximum intensity projections are shown in Figure 6 for both densities of each thickness of the auxetic patch. Both patch thicknesses and densities displayed even clustering distribution over the patch. The relative size of the cell clusters was larger in the high cell density groups with over 25 nuclei per cluster compared to low cell density groups with an average of 5 nuclei per cluster.

### 3.4. Calcium Handling of Induced Cardiomyocytes in Auxetic Patches

**Figure 7:**
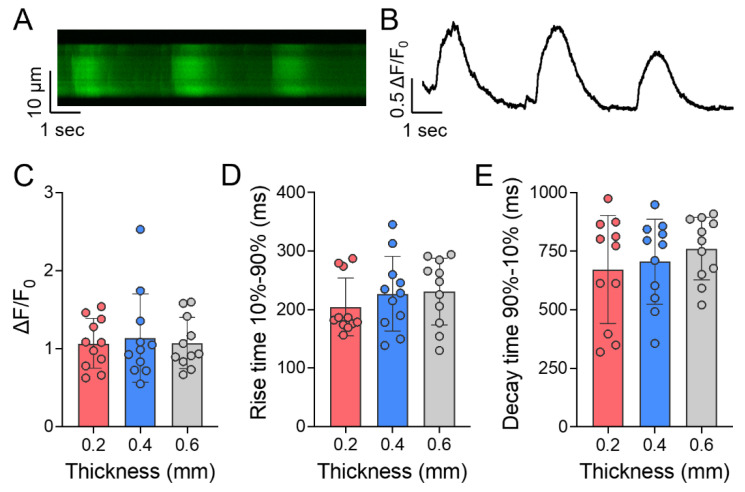
Calcium transients of day 7 high density iPSC-CM laden auxetic patches with three thicknesses. (**A**) Representative line scan from spontaneously beating iPSC-CMs in the patch; (**B**) Corresponding fluorescence trace; (**C**) Calcium transient amplitudes, (**D**) rise times, and (**E**) decay times for 0.2, 0.4, and 0.6 mm thickness patches. Mean ± SD.

Calcium transients were recorded from spontaneously beating high density iPSC-CMs to assess the calcium handling capacity of the cells embedded within the various thicknesses after 7 days of culture. A representative line scan and the corresponding fluorescence trace recorded from iPSC-CMs are shown in Figure 7A,B, respectively. Cells were exhibiting spontaneous beating activity under all the patch thicknesses assessed, however, no significant differences in the calcium transient amplitudes (Figure 7C), rise times (Figure 7D), or decay times (Figure 7E) of spontaneous calcium transients were observed between any of the groups.

## 4. Discussion

The ability of auxetic materials to stretch and match native heart tissue movement is promising for cardiovascular biomaterial designs. Previous work has developed a conductive auxetic cardiac patch that highlighted how auxetics demonstrate greater confirmation to local mechanics over non-auxetic patches, while also providing global mechanical support [16]. In the present study, we showed how multiple auxetic designs can be used to create tunable mechanics. We showed that a 0.2 mm orthogonal missing rib patch has comparable extension and tensile modulus to human heart physiology modulus reported, which varies from 0.02 to 0.5 MPa [10,11,12,13,14]. Due to this wide range of native cardiac values, increased thicknesses of orthogonal missing rib patches (0.2, 0.4, and 0.6 mm), for which extension ratio remained the same, were used to probe the impact of tensile modulus on cellular activity. For iPSC-CMs, substrates with physiological levels of stiffness have been shown to improve the electrical and mechanical outputs [30]. Although the addition of GelMA to the PCL substrate alters the tensile modulus slightly, incorporation of GelMA into a cardiac patch mimics the 3D native cardiac extracellular matrix-cell attachment profiles and has been previously shown to support cell viability and function [27,31,32]. The combination of GelMA and an auxetic PCL substrate creates a favorable microenvironment for cardiomyocyte cell culture that also could support synchronous active contraction and relaxation. While this biaxial phenomenon has not been previously examined, uniaxial patterned constructs have been shown to support unidirectional contractility and alignment [30,33]. Future investigation into long-term effects of auxetic scaffolds on iPSC-CMs could point to improved contractility, calcium handling, and transverse-tubule formation, as a more physiologically relevant model for native heart movement.

A more recent mechanical property consideration has been anisotropy, which is present in human and animal models. However, collagen content increases with time during the infarct remodeling period, so scar mechanical properties can vary structurally and mechanically based on this deposition through time [34]. For rat MI, a common animal model when evaluating cardiac patches, remodeling infarcts were found to be mechanically and structurally isotropic during all healing time points up to 6 weeks, irrelevant of fiber alignment and crosslinking [34]. By using this as a baseline for subsequent rat infarct model studies, our developed isotropic cardiac patch could provide local support for the infarcted region during the remodeling process. Future work will also focus on designing an anisotropic auxetic cardiac patch for advanced infarction models and comparing impact of isotropic and anisotropic auxetics on biological function.

We were able to create an auxetic patch composed of PCL, a biocompatible thermoplastic polymer that has been approved by the US Food and Drug Administration (FDA) for clinical use (K123633 FDA 510 k approval), and GelMA, which we showed maintains iPSC-CM phenotype and function at both a high and low cell density. The findings of this study further examine three thicknesses of the auxetic design and show that all groups can support basic cardiomyocyte function Our data for day 7 culture is comparable to previous studies [35,36], where induced cardiomyocyte spatial organization is relatively absent. These findings can be attributed to short culturing times and immaturity of the phenotype and cellular structures. At day 14, the organization of the induced cardiomyocytes into clusters suggests cell migration through the GelMA matrix for closer proximity to enable cell-to-cell communication. This phenomenon can be visualized over a 7-day period from 7 to 14 days of culture. A possible explanation could be that the first week of 3D culture is acclimation and the second week is eliciting a movement response. As the large clusters (>25 nuclei) are present with the higher seeding density of 100 M/mL, this indicates that the larger volume of cells in closer proximity may improve migration and aggregation of cardiomyocytes. Further quantitative studies are required to examine these parameters more closely over longer culture conditions with more intermittent sampling.

Added support through co-culture with endothelial cells and smooth muscle cells could be used enhance cardiomyocyte organization and function in future studies. Calcium transients on day 7 after cell seeding are comparable to previous literature studies of iPSC-CMs [36,37,38]. Our outcomes indicate that alterations in thickness, and as a result tensile geometric stiffness (as effective tangent modulus is constant over thickness), do not affect iPSC-CM function in short-term static culture, although cell viability is improved with thinner patches. To supplement this study, additional studies on long-term culture, co-culture with fibroblasts, and use of dynamic culture conditions could alter cell morphology and function. For example, environmental manipulation such as use of cyclic loading or electrical stimulation could lead to increased cardiomyocyte maturity and alignment as a function of biomaterial tensile modulus. Supplementary examination of electrical signal propagation using mature cardiomyocytes can demonstrate the potential for synchronous beating across the entire patch.

Expanded studies on long-term auxetic cardiomyocyte culture could identify improved cardiac therapeutical methods for treating myocardial infarction and other cardiovascular disease. The ability of auxetics to repeatedly expand or contract in axial and transverse directions when under uniaxial tension or compression also simplifies benchtop studies to use unidirectional equipment as opposed to biaxial setups. Another important consideration for infarct remodeling is expansion, which involves thinning and dilation of the ventricular walls over time. Although not investigated in this study, the auxetic pattern can improve the patch’s synclastic curvature, a valuable component of modeling the expansion zones. This allows auxetics to be used as a potential platform to examine cell culture, disease modeling, embryology, and new tissue engineering approaches [39].

## 5. Conclusions

Here, we designed a 3D printing based auxetic substrate that can be tuned to match heart mechanics based on variable auxetic patterns. An orthogonal missing rib auxetic was mechanically evaluated with three varying thicknesses of 0.2, 0.4, and 0.6 mm to simulate physiological ranges of myocardium tangent moduli, and the 0.2 mm patch was found to be within reported myocardium mechanical properties. We have demonstrated that an auxetic cardiac patch composed of PCL and GelMA can maintain induced cardiomyocyte functionality for 14-day culture. This study establishes the preliminary use of cellularized auxetic designs for cardiac patches and exploring their potential advantages when combined with iPSC-CMs for in vivo treatments of MI.

## Data Availability

All data are available from the corresponding authors upon reasonable request.

## Supplementary Materials

The following are available online at https://www.mdpi.com/article/10.3390/jcdd8120172/s1, Video S1: Cardiomyocyte beating during differentiation at day 11; Video S2: Cardiomyocyte beating in 0.4 mm cardiac patch after 7 days of culture; Figure S1: Cell viability following 7-day culture in auxetic cardiac patches. Figure S2: Immunofluorescent staining for α-actinin after day 1, 3, and 7 in the patches.

## References

[1] Virani S.S., Alonso A., Aparicio H.J., Benjamin E.J., Bittencourt M.S., Callaway C.W., Carson A.P., Chamberlain A.M., Cheng S., Delling F.N. (2021). Heart Disease and Stroke Statistics—2021 Update. Circulation.

[2] Frangogiannis N.G. (2017). The Extracellular Matrix in Myocardial Injury, Repair, and Remodeling. J. Clin. Investig..

[3] Nielsen S.H., Mouton A.J., DeLeon-Pennell K.Y., Genovese F., Karsdal M., Lindsey M.L. (2019). Understanding Cardiac Extracellular Matrix Remodeling to Develop Biomarkers of Myocardial Infarction Outcomes. Matrix Biol..

[4] Reis L.A., Chiu L.L.Y., Feric N., Fu L., Radisic M. (2016). Biomaterials in Myocardial Tissue Engineering. J. Tissue Eng. Regen. Med..

[5] Rienks M., Papageorgiou A.-P., Frangogiannis N.G., Heymans S. (2014). Myocardial Extracellular Matrix. Circ. Res..

[6] Jiang B., Yan L., Shamul J.G., Hakun M., He X. (2020). Stem Cell Therapy of Myocardial Infarction: A Promising Opportunity in Bioengineering. Adv. Ther..

[7] Ye L., Zimmermann W.-H., Garry D.J., Zhang J. (2013). Patching the Heart: Cardiac Repair from Within and Outside. Circ. Res..

[8] Feyen D.A.M., Gaetani R., Doevendans P.A., Sluijter J.P.G. (2016). Stem Cell-Based Therapy: Improving Myocardial Cell Delivery. Adv. Drug Deliv. Rev..

[9] Zhang J., Zhu W., Radisic M., Vunjak-Novakovic G. (2018). Can We Engineer a Human Cardiac Patch for Therapy?. Circ. Res..

[10] Silvestri A., Boffito M., Sartori S., Ciardelli G. (2013). Biomimetic Materials and Scaffolds for Myocardial Tissue Regeneration. Macromol. Biosci..

[11] Venugopal J.R., Prabhakaran M.P., Mukherjee S., Ravichandran R., Dan K., Ramakrishna S. (2012). Biomaterial Strategies for Alleviation of Myocardial Infarction. J. R. Soc. Interface.

[12] Chen Q.Z., Harding S.E., Ali N.N., Lyon A.R., Boccaccini A.R. (2008). Biomaterials in Cardiac Tissue Engineering: Ten Years of Research Survey. Mater. Sci. Eng. R Rep..

[13] Vunjak-Novakovic G., Tandon N., Godier A., Maidhof R., Marsano A., Martens T.P., Radisic M. (2010). Challenges in Cardiac Tissue Engineering. Tissue Eng. Part B Rev..

[14] Bouten C.V.C., Dankers P.Y.W., Driessen-Mol A., Pedron S., Brizard A.M.A., Baaijens F.P.T. (2011). Substrates for Cardiovascular Tissue Engineering. Adv. Drug Deliv. Rev..

[15] Streeter B.W., Xue J., Xia Y., Davis M.E. (2019). Electrospun Nanofiber-Based Patches for the Delivery of Cardiac Progenitor Cells. ACS Appl. Mater. Interfaces.

[16] Kapnisi M., Mansfield C., Marijon C., Guex A.G., Perbellini F., Bardi I., Humphrey E.J., Puetzer J.L., Mawad D., Koutsogeorgis D.C. (2018). Auxetic Cardiac Patches with Tunable Mechanical and Conductive Properties toward Treating Myocardial Infarction. Adv. Funct. Mater..

[17] Olvera D., Sohrabi Molina M., Hendy G., Monaghan M.G. (2020). Electroconductive Melt Electrowritten Patches Matching the Mechanical Anisotropy of Human Myocardium. Adv. Funct. Mater..

[18] Jin Y., Xie C., Gao Q., Zhou X., Li G., Du J., He Y. (2021). Fabrication of Multi-Scale and Tunable Auxetic Scaffolds for Tissue Engineering. Mater. Des..

[19] Wang Z., Luan C., Liao G., Liu J., Yao X., Fu J. (2020). Progress in Auxetic Mechanical Metamaterials: Structures, Characteristics, Manufacturing Methods, and Applications. Adv. Eng. Mater..

[20] Elipe J.C.Á., Lantada A.D. (2012). Comparative Study of Auxetic Geometries by Means of Computer-Aided Design and Engineering. Smart Mater. Struct..

[21] Maas S., Ellis B., Ateshian G., Weiss J. (2012). FEBio: Finite Elements for Biomechanics. J. Biomech. Eng..

[22] Maas S.A., Ateshian G.A., Weiss J.A. (2017). FEBio: History and Advances. Annu. Rev. Biomed. Eng..

[23] Buikema J.W., Lee S., Goodyer W.R., Maas R.G., Chirikian O., Li G., Miao Y., Paige S.L., Lee D., Wu H. (2020). Wnt Activation and Reduced Cell-Cell Contact Synergistically Induce Massive Expansion of Functional Human IPSC-Derived Cardiomyocytes. Cell Stem Cell.

[24] Lian X., Hsiao C., Wilson G., Zhu K., Hazeltine L.B., Azarin S.M., Raval K.K., Zhang J., Kamp T.J., Palecek S.P. (2012). Robust Cardiomyocyte Differentiation from Human Pluripotent Stem Cells via Temporal Modulation of Canonical Wnt Signaling. Proc. Natl. Acad. Sci. USA.

[25] Sharma A., Li G., Rajarajan K., Hamaguchi R., Burridge P.W., Wu S.M. (2015). Derivation of Highly Purified Cardiomyocytes from Human Induced Pluripotent Stem Cells Using Small Molecule-Modulated Differentiation and Subsequent Glucose Starvation. J. Vis. Exp..

[26] Lian X., Zhang J., Azarin S.M., Zhu K., Hazeltine L.B., Bao X., Hsiao C., Kamp T.J., Palecek S.P. (2013). Directed Cardiomyocyte Differentiation from Human Pluripotent Stem Cells by Modulating Wnt/β-Catenin Signaling under Fully Defined Conditions. Nat. Protoc..

[27] Bejleri D., Streeter B.W., Nachlas A.L., Brown M.E., Gaetani R., Christman K.L., Davis M.E. (2018). A Bioprinted Cardiac Patch Composed of Cardiac-Specific Extracellular Matrix and Progenitor Cells for Heart Repair. Adv. Healthc. Mater..

[28] Wang Z., Kumar H., Tian Z., Jin X., Holzman J.F., Menard F., Kim K. (2018). Visible Light Photoinitiation of Cell-Adhesive Gelatin Methacryloyl Hydrogels for Stereolithography 3D Bioprinting. ACS Appl. Mater. Interfaces.

[29] Bahney C.S., Lujan T.J., Hsu C.W., Bottlang M., West J.L., Johnstone B. (2011). Visible Light Photoinitiation of Mesenchymal Stem Cell-Laden Bioresponsive Hydrogels. Eur. Cells Mater..

[30] Ribeiro A.J.S., Ang Y.-S., Fu J.-D., Rivas R.N., Mohamed T.M.A., Higgs G.C., Srivastava D., Pruitt B.L. (2015). Contractility of Single Cardiomyocytes Differentiated from Pluripotent Stem Cells Depends on Physiological Shape and Substrate Stiffness. Proc. Natl. Acad. Sci. USA.

[31] Nichol J., Koshy S., Bae H., Hwang C., Yamanlar S., Khademhosseini A. (2010). Cell-Laden Microengineered Gelatin Methacrylate Hydrogels. Biomaterials.

[32] Koti P., Muselimyan N., Mirdamadi E., Asfour H., Sarvazyan N. (2019). Use of GelMA for 3D Printing of Cardiac Myocytes and Fibroblasts. J. 3D Print. Med..

[33] Huebsch N., Loskill P., Deveshwar N., Spencer C.I., Judge L.M., Mandegar M.A., Fox C., Mohamed T.M.A., Ma Z., Mathur A. (2016). Miniaturized IPS-Cell-Derived Cardiac Muscles for Physiologically Relevant Drug Response Analyses. Sci. Rep..

[34] Fomovsky G.M., Holmes J.W. (2010). Evolution of Scar Structure, Mechanics, and Ventricular Function after Myocardial Infarction in the Rat. Am. J. Physiol.-Heart Circ. Physiol..

[35] Maiullari F., Costantini M., Milan M., Pace V., Chirivì M., Maiullari S., Rainer A., Baci D., Marei H.E.-S., Seliktar D. (2018). A Multi-Cellular 3D Bioprinting Approach for Vascularized Heart Tissue Engineering Based on HUVECs and IPSC-Derived Cardiomyocytes. Sci. Rep..

[36] Cui H., Liu C., Esworthy T., Huang Y., Yu Z.-X., Zhou X., San H., Lee S.-J., Hann S.Y., Boehm M. (2020). 4D Physiologically Adaptable Cardiac Patch: A 4-Month in Vivo Study for the Treatment of Myocardial Infarction. Sci. Adv..

[37] Gao L., Kupfer M.E., Jung J.P., Yang L., Zhang P., Da Sie Y., Tran Q., Ajeti V., Freeman B.T., Fast V.G. (2017). Myocardial Tissue Engineering with Cells Derived from Human-Induced Pluripotent Stem Cells and a Native-Like, High-Resolution, 3-Dimensionally Printed Scaffold. Circ. Res..

[38] Hwang H.S., Kryshtal D.O., Feaster T.K., Sánchez-Freire V., Zhang J., Kamp T.J., Hong C.C., Wu J.C., Knollmann B.C. (2015). Comparable Calcium Handling of Human IPSC-Derived Cardiomyocytes Generated by Multiple Laboratories. J. Mol. Cell. Cardiol..

[39] Mardling P., Alderson A., Jordan-Mahy N., Le Maitre C.L. (2020). The Use of Auxetic Materials in Tissue Engineering. Biomater. Sci..

