# Supplementary material for: Designing a 3D Printing Based Auxetic Cardiac Patch with hiPSC-CMs for Heart Repair
Source: J Cardiovasc Dev Dis. 2021 Dec 3;8(12):172. doi: 10.3390/jcdd8120172 (PMC8706296; doi:10.3390/jcdd8120172)
Supplement: Supplementary file 1 [file jcdd-08-00172-s001.zip › Figure S1-S2.pdf]

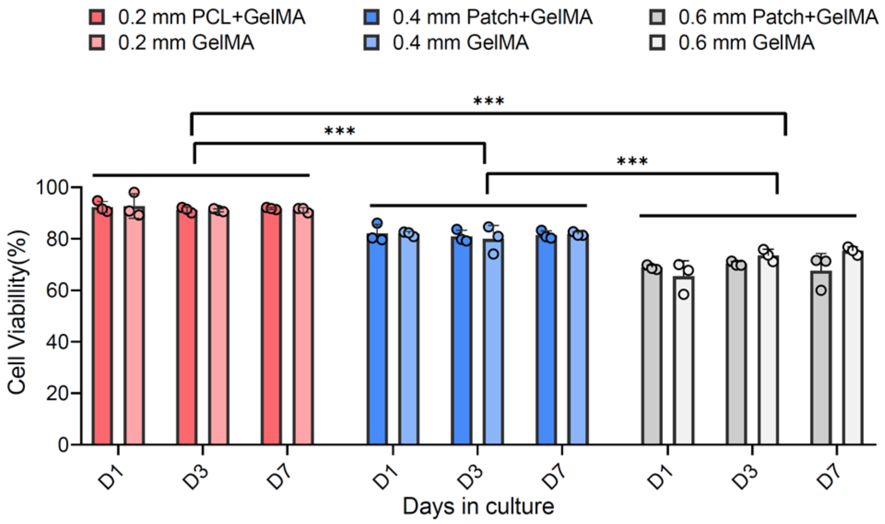

Figure S1:Cell viability following 7-day culture in auxetic cardiac patches.

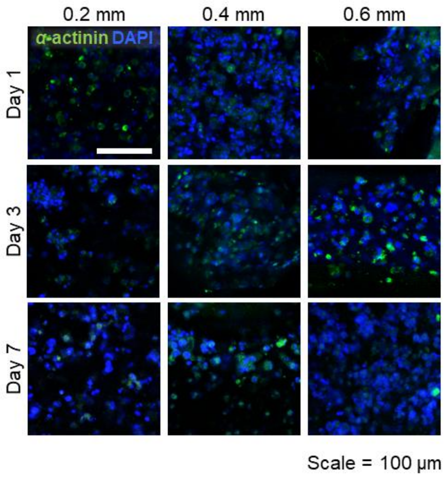

Figure S2: Immunofluorescent staining for α-actinin after day 1, 3, and 7 in the patches.
